# AutoScanJ: A Suite of ImageJ Scripts for Intelligent Microscopy

**DOI:** 10.3389/fbinf.2021.627626

**Published:** 2021-03-18

**Authors:** Sébastien Tosi, Anna Lladó, Lídia Bardia, Elena Rebollo, Anna Godo, Petra Stockinger, Julien Colombelli

**Affiliations:** ^1^ Institute for Research in Biomedicine, IRB Barcelona, Barcelona Institute of Science and Technology, BIST, Barcelona, Spain; ^2^ Molecular Imaging Platform, Molecular Biology institute of Barcelona IBMB-CSIC, Barcelona, Spain; ^3^ Genetics of Male Fertility Group, Cell Biology Unit, Faculty of Biosciences, Autonomous University of Barcelona, Bellaterra, Spain; ^4^ Center for Genomic Regulation, CRG, Barcelona Institute of Science and Technology, BIST, Barcelona, Spain

**Keywords:** light microscopy, bioimage analysis, intelligent microscopy, imagej, micro-manager, events detection, objects detection

## Abstract

We developed AutoscanJ, a suite of ImageJ scripts enabling to image targets of interest by automatically driving a motorized microscope at the corresponding locations. For live samples, our software can sequentially detect biological events from their onset and further image them at high resolution, an action that would be impractical by user operation. For fixed samples, the software can dramatically reduce the amount of data acquired and the acquisition duration in situations where statistically few targets of interest are observed per field of view. AutoScanJ is compatible with motorized fluorescence microscopes controlled by Leica LAS AF/X or Micro-Manager. The software is straightforward to set up and new custom image analysis workflows to detect targets of interest can be simply implemented and shared with minimal efforts as independent ImageJ macro functions. We illustrate five different application scenarios with the system ranging from samples fixed on micropatterned surfaces to live cells undergoing several rounds of division. The target detection functions for these applications are provided and can be used as a starting point and a source of inspiration for new applications. Overall, AutoScanJ helps to optimize microscope usage by autonomous operation, and it opens up new experimental avenues by enabling the real-time detection and selective imaging of transient events in live microscopy.

## Introduction

High-resolution fluorescence microscopy can generate an overwhelming amount of data and require a prohibitive acquisition time when imaging a wide sample area. Additionally, for live microscopy, guaranteeing sample integrity also puts hard limits on acquisition speed and maximum light dose. Fortunately, it often turns out that only part of the data acquired is useful to an experimenter interested in rare events or sparsely spread objects. Image acquisition can then naturally be re-organized in two sequential scans: 1) a scan covering a large sample area with resolution adjusted to allow the reliable detection of the targets of interest (primary scan) and 2) a higher resolution scan with fields of views selectively centered on the targets of interest (secondary scan). We refer to this technique as *Intelligent Microscopy* [a.k.a *Intelligent Acquisition* (Micro-Manager, 2020) or *Online Feedback Analysis Microscopy* (Zeiss, 2020)].

The proprietary acquisition software of commercial microscopes now often offer advanced protocols combining imaging modalities, *mosaic* scans, high content screening, and partial support for Intelligent Microscopy (Leica, 2020; Nikon, 2020; Zeiss, 2020). In practice however, the inherent fragmentation of the market makes it difficult to share reproducible Intelligent Microscopy protocols across laboratories, and no turnkey solution is currently provided for the selective imaging of events of interest in live samples. Open source software for image acquisition (Edelstein et al., 2010) and image analysis (de Chaumont et al., 2012; Schneider et al., 2012; McQuin et al., 2018; Berg et al., 2019) are overall more interoperable and easier to deploy anywhere. For instance, Micro-Manager (Edelstein et al., 2010) is a versatile open source software to control camera-based microscopes which naturally integrates with ImageJ, one of the most widely used bioimage analysis software. Micro-Manager offers two plugins (Pinkard et al., 2016; Micro-Manager, 2020) to perform Intelligent Microscopy. The first one is designed to selectively image objects of interest in fixed samples while the second can map a 3D sample at coarse resolution and restrict posterior image acquisition (e.g. only to sample surface) with the target acquisition settings for the application.

AutoScanJ is compatible with Micro-Manager (Supplementary Data S0.0) and it brings a new dimension to Intelligent Microscopy by enabling the real-time detection of events of interest in live samples. For increased flexibility, the software is also compatible with Leica confocal microscopes^1^ (Supplementary Data S7.1). Several acquisition modes are supported to adapt to the organization of the sample, and the Image analysis workflows used for target detection of new applications can be simply developed and loaded as ImageJ macro functions independent from the main software. Finally, the client-server architecture of the software enables offloading particularly demanding image analysis to a dedicated distant workstation over a network. We illustrate this flexibility for a broad range of applications both for fixed and live samples.

## Method

**FIGURE 1:**
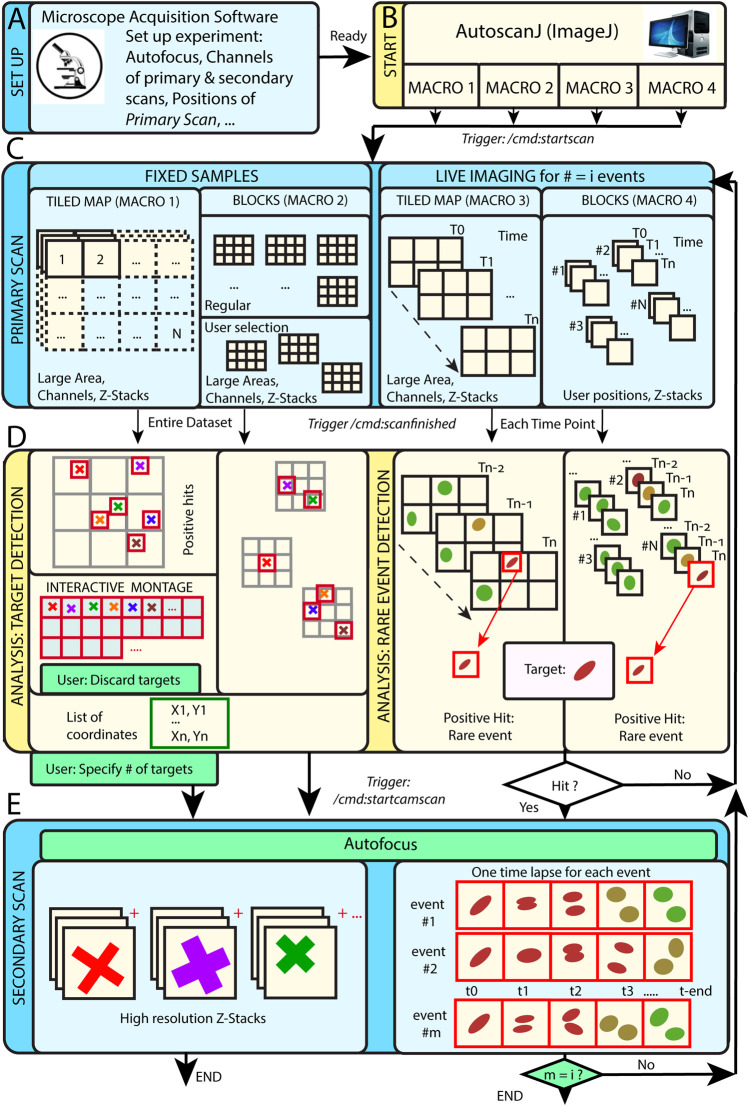
AutoScanJ acquisition modes for fixed and live samples experiments. **(A)** The microscope’s parameters are set by the user before launching AutoscanJ, in particular the starting position(s) of the “Primary Scan”. **(B)** AutoscanJ is run with one of four modalities by launching an ImageJ macro of choice. Commands are sent through the network (vertical arrows between boxes) when using a remote workstation for processing. **(C)** The sample is monitored by periodically tiling a large area at low resolution (primary scan). For fixed samples **(Left)**, a single Tiled Map (i.e. Mosaic large area) or multiple Tiled maps (“Blocks”) are scanned, while for Live Imaging **(Right)** a Tiled map or user selected positions are continuously acquired. **(D)** The images acquired are analyzed to find events of interest. For fixed samples **(Left)**, the entire datasets are processed to detect targets in the images. The single Tiled Map returns an interactive montage displaying all targets detected in a tight grid, where the user can optionally discard targets (i.e. false positive ones) and set a fixed number to be acquired. A list of X,Y coordinates is built and used for the secondary scan. For Live Imaging **(Right)**, AutoscanJ must process at least three time points to detect rare events, and the primary scan runs “on the fly” until a “hit” is found, which triggers the secondary scan for a defined duration. **(E)** Secondary scan: in Live mode, when an event is detected the microscope switches to secondary scan to acquire a fixed length high-resolution time-lapse centered on the detected event. After this, the microscope switches back to primary scan monitoring until the next event is detected and AutoscanJ stops after the user-defined number of events is detected. With fixed samples, AutoscanJ stops after the secondary scan. Light blue boxes: Microscope operations, Light yellow boxes: AutoscanJ, image analysis workstation operations, Green boxes: User interventions (optional).

AutoScanJ is distributed as a suite of four ImageJ macros corresponding to the possible combinations of these acquisition scenarios: 1) fixed or live sample and 2) continuous mosaic (tiled map) or regular/freely defined acquisition locations (block mode), as illustrated in Figure 1. The software, documentation and test data can be found at: https://github.com/SebastienTs/AutoScanJ.

### Tiled Map

This operation mode is useful to monitor large regions of a sample by acquiring adjacent image tiles (Figure 1C). After acquisition, the tiles are automatically laid side by side, maximum intensity projected, and the primary scan map is handed to a custom image analysis function (Figure 1D). This function is expected to return a list of target coordinates (ImageJ multi-point selection) that is subsequently sent to the acquisition software to sequentially acquire higher resolution images of the targets during the secondary scan (Figure 1E).

**FIGURE 2:**
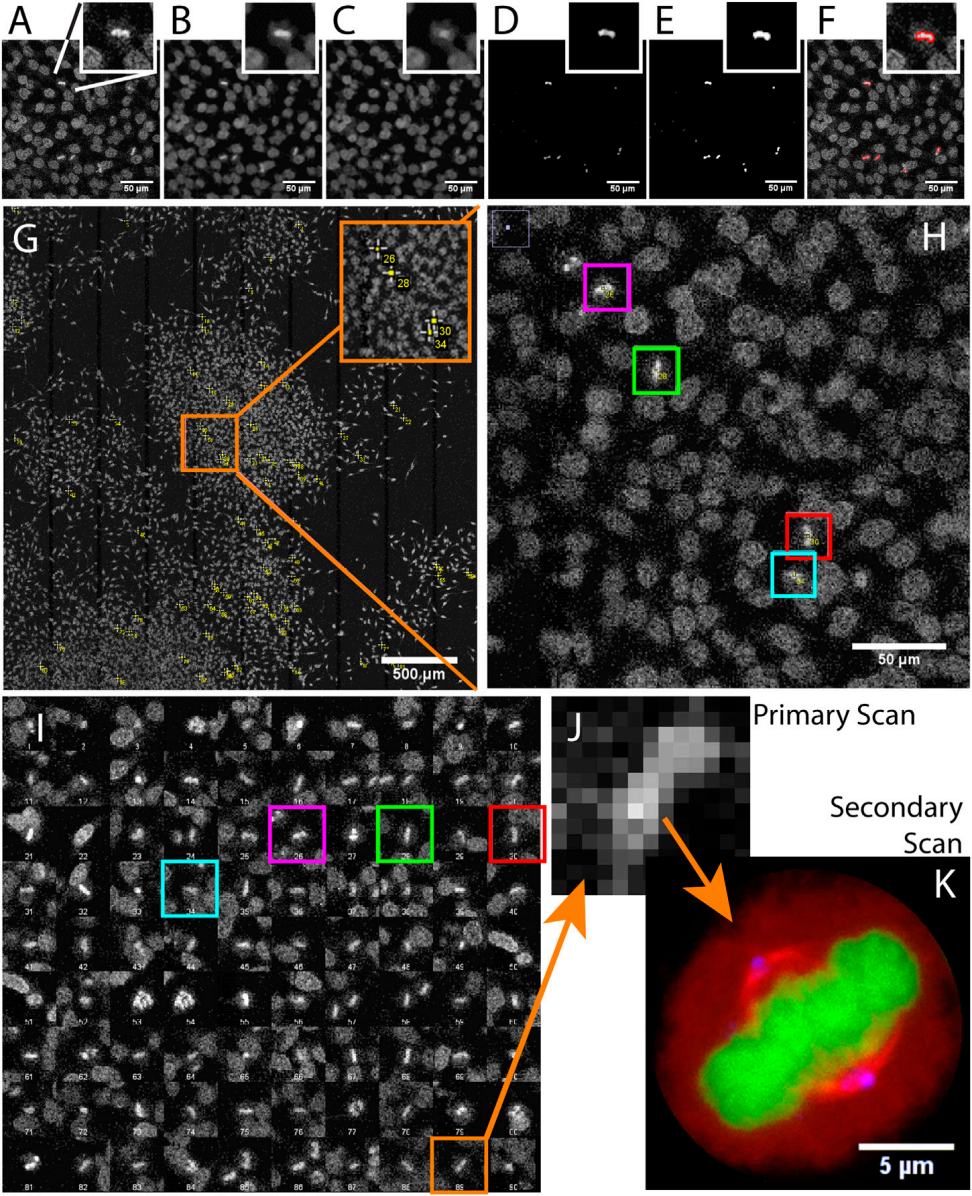
Acquiring mitotic cells at high resolution with AutoScanJ (confocal fluorescence microscopy). A to F Image analysis workflow with a cropped region from the primary scan map and a single mitotic cell closer view (inset). **(A)** Raw image, **(B)** Median filter, **(C)** ImageJ *Remove outliers*, **(D)** Subtracting image C to image B, **(E)** Thresholding non-zero pixels, **(F)** Connected particles of area greater or equal than 12 pixels (in red) overlaid over image A. **(G)** Mitotic cells detected during primary scan (yellow crosses from ImageJ, enhanced in inset). **(H)** Zoomed area with four mitotic cells (colour boxes).**(I)** Interactive montage of all detected mitotic cells, including corresponding colour boxes of H]. **(J)** Zoomed image of a detected mitotic cell (primary scan) and **(K)** The same cell acquired during the secondary scan, DNA (green), tubulin (red) and centrosomes (blue). All the images shown are z-stacks maximum intensity projections. Scale bars: A–F, H - 50 µm, G - 500 µm, H - 50 µm, K - 5 µm.

For fixed samples, the primary map is acquired only once and the target selection can optionally be validated and refined by the user from an interactive montage (Figure 1D, Figure 2I) showing cropped regions around the targets. It is also possible to limit the number of targets acquired during the secondary scan (Supplementary Data S0.2).

For live samples, primary scan maps are periodically acquired (Figure 1C), and the maps from the three last time frames are automatically queued and handed in to the target detection function. When a target of interest is detected in a given time frame, the system immediately switches to secondary scan (without user validation) and it acquires a fixed-length time-lapse centered on the detected target before switching back to primary scan (until the next target detection). In effect, the system is consequently “blind” to new events occurring while acquiring secondary scan images (until switching back to primary scan mode) and optimized for fast live imaging of single targets.

### Block Mode

In block mode, during the primary scan, the images are acquired as independent fields of view (or small tiled grids) centered around regularly spaced (or user defined) positions (Figure 1C). This mode is to be favored when the sample naturally shows a regular organization [e.g. multi-well plates, micropatterned cells (Théry, 2010)], to detect events from slowly moving objects in live experiments, or if the amount of data acquired during the primary scan is too large to be comfortably processed as a single batch in the memory of the workstation.

For fixed samples, all the blocks are analyzed sequentially (Figure 1D), targets are accumulated to a list, and a single secondary scan is launched to acquire all targets once all the blocks have been analyzed. Target refinement is usually disabled since typically many targets are detected and their visual inspection would be too demanding. It is however possible to do it, for instance for debugging purposes, or to only check the first blocks of an experiment.

For live samples, all the blocks are continuously monitored during primary scan and the software switches to secondary scan (Figure 1E) immediately after an event is detected in the block currently analyzed (Figure 1D). The primary scan monitoring of all the blocks is only resumed after acquiring a fixed-length time-lapse centered on the detected event.

### Client-Server and *Offline* Operation

AutoScanJ is built around a client-server architecture (Supplementary Data S0.1), which makes it very easy to offload heavy computations to a dedicated image analysis workstation. The communication between AutoScanJ macro and the acquisition software (Micro-Manager or Leica LAS AF/X) relies on a subset of Leica CAM protocol network commands (Supplementary Data S0.3), and the image files are exchanged through a network folder. However, unless the image analysis task at hand is really demanding (e.g. high memory requirements), it is possible to use a single workstation both to control the microscope and to analyze the images. The software can also conveniently run in *offline* mode, by using previously acquired primary scan images (Supplementary Data S2). This can be useful to test the software without a microscope connected, or to debug the target detection image analysis workflow after having acquired some representative primary scan images (Supplementary Data S5).

## Results

### Imaging Mitotic Cells (Tiled Map/Fixed Sample)

We used AutoScanJ to perform Intelligent Microscopy of asynchronously dividing HeLa cells stained with DAPI nuclear DNA dye (Supplementary Data S6-M1). During the primary scan, a low resolution tiled map of 10 x 10 images was acquired (Supplementary Data S2-D1) and analyzed by a custom ImageJ macro to identify sparsely spread mitotic cells based on the increased fluorescence from DNA condensation (Figure 2). This ImageJ macro is available as ***_Metaphase_detector* AutoScanJ** sample workflow. The mitotic cells were then reviewed by the user from a montage generated by the software (Figure 2I) before acquiring these cells at higher resolution during the secondary scan (Figure 2K). The image resolution was dramatically lower during the primary scan than during the secondary scan (Figure 2J), but still sufficient to enable the reliable detection of mitotic cells. The same objective lens was used for both scans but the primary scan was greatly accelerated by decreasing the confocal zoom, the number of pixels per field of view and the number of Z slices acquired (with larger pinhole). Also, a single fluorescence channel (DAPI) was acquired during the primary scan while multiple channels were acquired during the secondary scan.

For this application, 900 image tiles would have been required to cover the primary scan sample region at the resolution of the secondary scan. Each tile would have held 195 image slices (3 channels, 65 Z slices), totaling 175.500 images (171.4 GB). In contrast only 1.5 GB was acquired with AutoScanJ for the same relevant content to the study, and the whole experiment could be performed in less than 2 h. This is about two orders of magnitudes less than the time that would have been required to acquire the same sample region with the secondary scan acquisition settings.

### *Fishing* Nuclei With Abnormal FISH Signature (Tiled Map/Fixed Sample)

**FIGURE 3:**
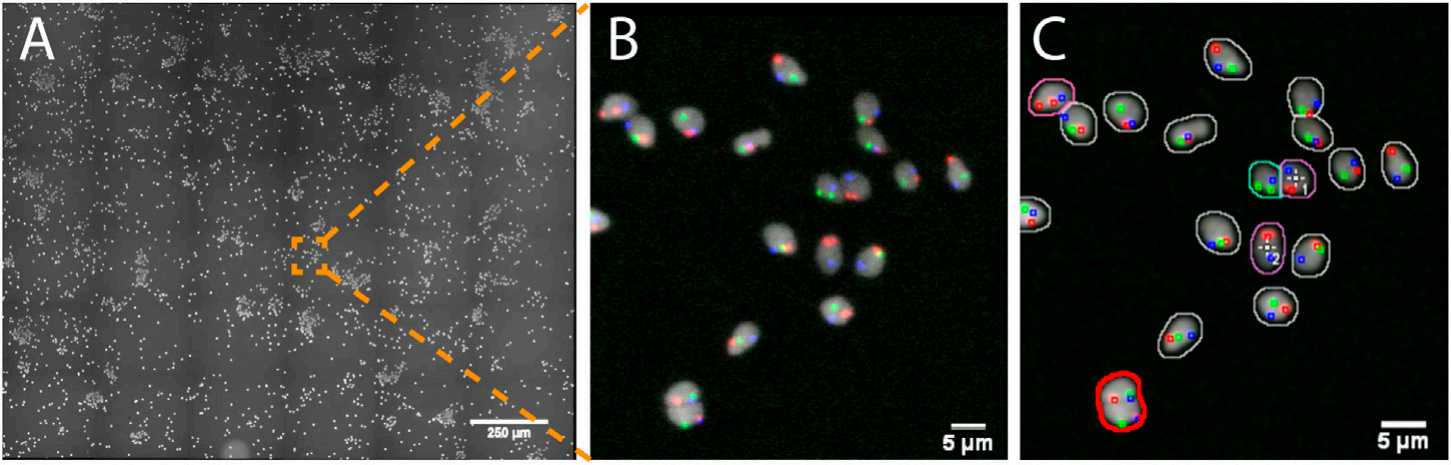
Imaging spermatozoa exhibiting a specific abnormal genotype with AutoScanJ (widefield fluorescence microscopy). **(A)** Primary scan map (DAPI channel). **(B)** Zoomed area showing DAPI (white) and three FISH channels (red, green, blue). **(C)** Same image with an overlay displaying the nuclei and FISH signals detected. The nuclei contours are color coded based on their FISH content and the cells missing the chromosome associated with the green FISH signal are detected as targets of interest (white crosses). Nuclei circled in red are excluded due to their invalid shape or size. All the images shown are z-stacks maximum intensity projections. Scale bars: A- 250 µm, B-C 5 µm. See Supplementary Material for secondary scan images.

In this study, human spermatozoa exhibiting abnormal genotypes (e.g. the presence or absence of specific chromosomes) were studied from Fluorescence *in situ* Hybridization (FISH) probes labeling the chromosomes of interest (e.g. X, Y and 18, Supplementary Data S6-M2). The project aimed at acquiring high resolution 3D images of nuclei showing a set of abnormal phenotypes so as to analyze the location of the chromosomes within these abnormal nuclei. Due to the low occurrence of some abnormal genotypes (e.g. less than 1%) tens of thousands of spermatozoa would theoretically have to be acquired at high resolution so that a sufficient number of abnormal spermatozoa could be studied. To reduce the amount of data collected, we used AutoScanJ to 1) find abnormal spermatozoa from a low resolution primary scan (Figure 3) and 2) selectively acquire them at higher resolution in order to study chromosome territories inside the nuclei (Supplementary Figure S3.3 and Supplementary video S1-V7). Abnormal spermatozoa were detected from a primary scan covering a large region of the sample. The detection was performed from the nuclear label and aimed at segmenting isolated (or confidently split) nuclei and classifying them based on the FISH signals that were detected inside. The target detection function supported the detection of multiple phenotypes during the same experiment (Supplementary Data S3.3). To ensure a fast primary scan at a resolution adapted to the reliable detection of the FISH signals, a widefield microscope with optimized filter wheels was employed (Supplementary Data S7.2). The speed of the primary scan was further increased by using camera binning and by limiting the number of Z slices acquired to the bare minimum (three slices). This was enabled by first estimating a coarse focus map interpolated from the corners of the regions acquired and then running Micro-Manager Autofocus prior to acquiring each secondary scan target (Supplementary Data S0.2). Primary scans were typically completed in less than 1 h and could be analyzed in only a few minutes by the target detection function we developed, while several days would have probably been required by a trained experimenter to reliably mark all abnormal nuclei. An added advantage of AutoScanJ is that all detected targets could be inspected side by side at a glance, which greatly helped their validation and refinement. Secondary scans were launched overnight and typically yielded up to fifty high resolution image stacks of abnormal spermatozoa, that were used to study the accurate 3D localizations of the chromosomes in the nuclei (chromosome territories) as normalized longitudinal (xy) and radial (xz) positioning inside sperm nuclei. AutoScanJ also makes it possible to re-acquire the same secondary targets multiple times. This could for instance be useful to perform rounds of washing and staining of the same sample to obtain richer information (Supplementary Data S0.2).

### Studying Isolated Cells on Fibronectin Micropatterned Surfaces (Block/Fixed sample)

**FIGURE 4:**
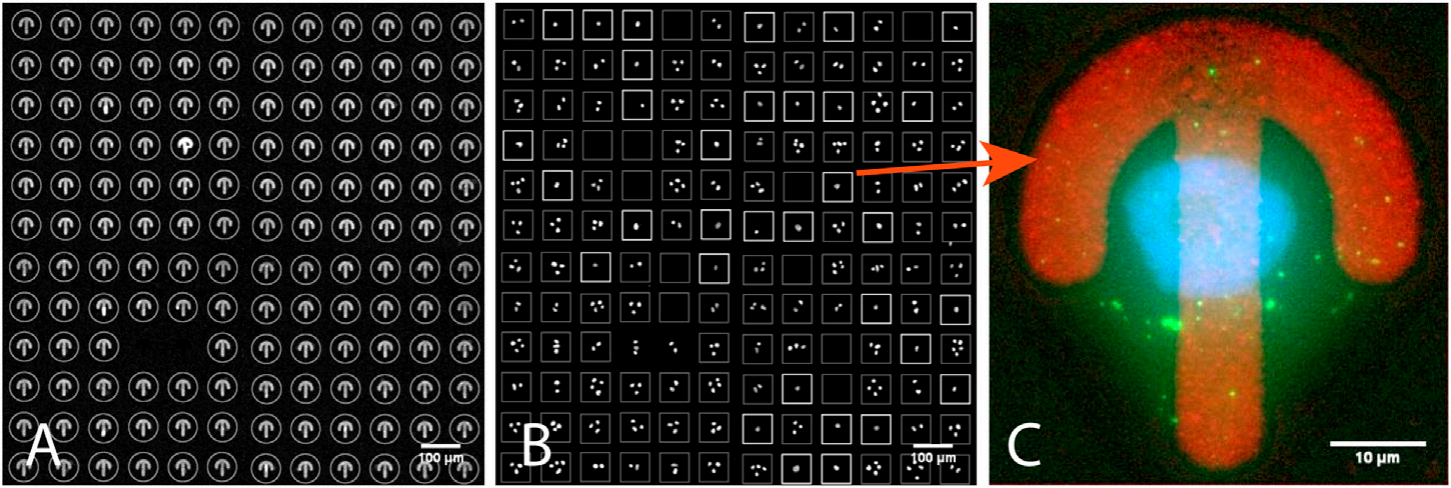
One of the 144 blocks of a CYTOO *starter chip* (confocal fluorescence microscopy). **(A)** Detected micropatterns in Fibronectin-Alexa 647 channel circled in gray. **(B)** DAPI channel: white boxes correspond to detected isolated nuclei showing individual cells on patterns; dimmer boxes are patterns with no or more than one nucleus. **(C)** An image from the secondary scan showing a single-cell-on-pattern HeLa cell expressing GFP-LC3 and stained with DAPI. All the images shown are z-stacks maximum intensity projections. Scale bars: A,B - 100 µm, C - 10 µm.

In this application we were interested in finding single isolated HeLa cells sitting on Fibronectin micropatterned surfaces (Supplementary Data S6-M3). The micropatterns CYTOO starter chip (Théry, 2010) used for this experiment was made of 144 blocks, each holding a grid of 12x12 patterns with different shapes (four different shapes and three different sizes). We used it as a proof of concept to demonstrate the automated imaging and processing of whole CYTOO chips, and report single cell pattern frequency for varying pattern size (final statistics not shown in this study). The output of this experiment could also be used as the basis to build an *average cell* atlas (Théry, 2010) in a fully automated fashion, for instance to study how the geometry of the patterns influences cell polarization. In our hands, even for optimized cell seeding conditions, only a few patterns featured a single cell, most patterns being either empty or holding more than one cell. Scanning the whole chip at high resolution would hence be very inefficient. Accordingly, AutoScanJ was employed and low resolution images of every block were acquired during the primary scan. The image analysis workflow was designed to first localize valid patterns from their fluorescent label channel (Figure 4A **)**, and then analyze the content of the nuclear channel inside a square bounding box circling valid patterns (Figure 4B). The corresponding image analysis workflow is provided as ***_Cytoo_Isolated_Nucleus_Confocal*** AutoScanJ sample target function. To determine if a single cell was sitting on a given pattern, significant intensity minima were counted inside the associated pattern bounding box after enhancing the nuclei by Laplacian of Gaussian filtering of the DAPI images (Supplementary Data S3.1). Only the bounding boxes holding exactly one nucleus were selected and accumulated to a list of positions acquired during the secondary scan (Figure 4C). Since only a single micropattern could be imaged per field of view at the target resolution, 20.736 (144 blocks x 144 patterns) fields of view (each a 75 image stack) would be required to cover the whole chip, totaling 1.555.200 images (388.8 GB). In practice, the cell seeding density yielded an average of about 15% isolated cells per block. The total amount of data acquired could then be theoretically reduced to some 58 GB. In practice, only a fixed number of cells (typically 100) were acquired, further reducing the amount of data. Even then, AutoScanJ provided a helpful overview of single cell occupancy over the whole chip, which helped optimizing the seeding density. Sample data from the primary scan imaging of four CYTOO blocks are available for testing *offline* (Supplementary Data S2-D2).

### Acquiring Mitosis Events From Their Onset (Tiled map/Live sample)

**FIGURE 5:**
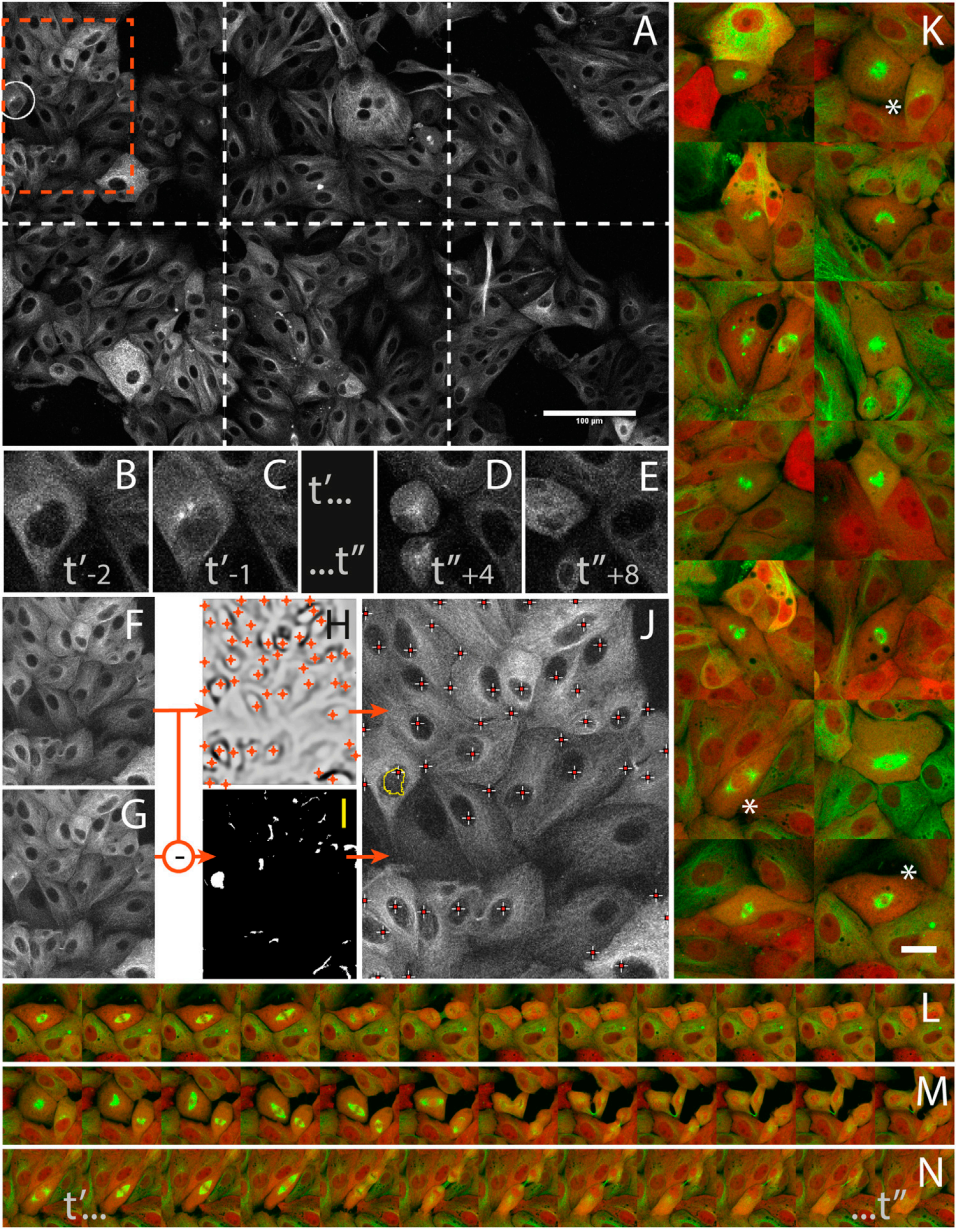
Capturing mitosis events from their onsets with AutoScanJ (LLC-PK cells, α-tubulin, confocal fluorescence microscopy). **(A)** A frame (at time t’_-1_) of the 3x2 tiled primary scan map showing a positive event at the onset of a mitosis is detected (white circle). **(B,C)** time sequence of the detected mitotic cell before switching to secondary scan: t’ and t’’ are start- and end-time points of secondary scan, indices are frame numbers. **(D,E)** The same cell after mitosis in the next primary scan. **(F–J)**] Associated image analysis workflow (cropped region from orange box in A] including the mitotic cell). Frame t’_-2_ **(F)** is subtracted to frame t’_-1_ **(G)**, and the result is median-filtered and thresholded **(I)**. Particles resembling a nucleus in size and shape (see the largest particle in I) are added to a pool of candidates, and validated as “detected mitosis” if a nucleus was detected inside (see crosses in J) in frame t_−2_. Nuclei are detected as local intensity minima from frame t_-2_ LoG filtered image (H, shown with inverted contrast), and reported as white/red crosses in final image **(J)**. K] A montage of secondary scan images from 14 mitotic cells (green: α-tubulin, red: Centrin) detected during a 16 h long experiment. The same first time frame at t’ is represented for all mitoses. **(L–N)** Three selected time lapse sequences of the secondary scan from t’ to t’, acquired every 5 min. All images shown are z-stacks maximum intensity projections. Scale bars: A - 100 µm, K - 20 µm.

LLC-PK cell division was studied from high resolution time-lapses capturing mitosis events from their onsets. The cells were stably expressing mCherry-α-tubulin and GFP-Centrin (Supplementary Data S6-M4) and mitosis onset was identified by detecting the nuclear membrane breakdown when α-tubulin invades the space formerly occupied by the nucleus to interlace with chromatin (Figures 5B,C) right before mitosis (Figures 5D,E). During a 16 h operator-free experiment, an extended region of the sample (Figure 5A) was monitored by periodic primary scans (Supplementary Data S2-D4). During this experiment, 14 mitosis events could be detected (Supplementary data S1-V1 and S1-V2) by the image analysis workflow described in Figures 5F–J, and no incorrect event was detected and acquired during the secondary scan. Each time, a 1 h high-resolution time-lapse of the mitosis was successfully recorded around the detected positions (Figure 5K **)**. The microscope was hence imaging at the target resolution about 90% of the time while it was monitoring the sample in primary scan about 10% of the time.

The same kind of experiment was also performed using *Drosophila* neuroblast primary cultures (Supplementary Data S6-M5). This time, a nuclear marker (His2Av) was used to detect the asymmetric division of neuroblasts into daughter cells (Supplementary Data S1-V3, S1-V4, S1-V5 and S1-V6) and the primary scan was set as 12 independent fields of view (block mode), initially centered by the user around neuroblasts and simultaneously monitored during the experiment. The image analysis workflow used for target detection is relatively simpler than for the previous application but due to the very high motility of the sample, great care was taken in designing a mechanism to limit the false positive rate of target detections (Supplementary Data S3.2). Despite this, the false positive rate was found around 25%, but it could probably be greatly reduced by relying on properly trained deep learning networks such as U-Nets (Ronneberger et al., 2015).

## Discussion

While it might be practically feasible to inspect a fixed sample through the oculars of a microscope and manually mark a few positions of interest, this is virtually impossible to achieve when thousands of objects have to be carefully inspected individually, or when transient events have to be quickly spotted in live samples. We have demonstrated the capability of AutoScanJ to address these two challenges for five applications performed in the context of various scientific projects.

Most microscope manufacturers nowadays offer integrated tools for complex acquisition protocols including support for image analysis feedback, but these tools only provide turnkey solutions for some fixed samples experiments, and they are invariably sold as add-on modules with significant price tag. Additionally, image analysis is bound to proprietary modules (Nikon, 2020; Zeiss, 2020), which compromises reproducibility and limits flexibility. Open source solutions are available for Intelligent Microscopy but they either consist in charting a 2D (Carro et al., 2015) or 3D (Peravali et al., 2011; Pinkard et al., 2016) sample so as to restrict the imaging area, or they are designed for target detection from still images (Tischer et al., 2014; Booth et al., 2018; Micro-Manager, 2020) (even though live microscopy can be performed at the detected positions). An exception is Micro-pilot (Conrad et al., 2011), a pioneering project designed for the real-time detection of cell mitosis (and their subsequent FRAP manipulation), but this software is difficult to adapt to other applications since it was crafted for this specific application and developed in a low level language for a set of very specific microscopes. Other related feedback microscopy projects (see Supplementary Table S1) either aim at detecting events from live microscopy time-lapses to trigger hardware devices (Almada et al., 2019), track moving objects/regions to adjust the acquisition position (Rabut and Ellenberg, 2004), or perform optogenetics photo-manipulation (Leifer et al., 2011).

In turn, AutoScanJ is intended to democratize the access to Intelligent Microscopy. It is built around the paradigm of primary and secondary scans triggered from target detection and it aims at reusability, flexibility and versatility (Supplementary Table S1). This is achieved by supporting two microscope control platforms (Micro-Manager and Leica LAS AF/X) able to control all main microscopy modalities, by supporting both fixed and live target detection and two acquisition modes (tiled region or regular/user defined positions), by enabling user intervention to refine target detection, and by supporting a client-server architecture to perform offload demanding image analysis to a dedicated workstation. AutoscanJ is easy to set up, fully documented (Supplementary Data S4), relies on the simple ImageJ macro language for target detection and it comes with four primary scan sample datasets (Supplementary Data S2) and associated image analysis workflows that aim to bring a good starting point and source of inspiration to tackle developing new target detection function for other applications (Supplementary Data S5). To further help users getting started, we also provide a target detection function detecting targets in tissues, e.g. kidney glomeruli, that can be easily reproduced by using the same commercial sample slide (Supplementary Data S2-D3) and similar imaging conditions (Supplementary Data S6-M6). Finally, AutoScanJ is very modular since target detection functions can be easily appended to independent macro files (one for each of the four possible scenarios), which are automatically registered and made available from AutoScanJ main dialogue box.

### Perspectives

Even though AutoScanJ already covers a wide range of acquisition modalities, some useful applications tackled by other software are not supported (Supplementary Table S1). To remedy this, we especially plan a new AutoScanJ modality (ImageJ macro) for which targets are detected from still images during the primary scan and then subsequently and cyclically imaged during interleaved parallel secondary time-lapses. We also plan a tighter integration with Micro-Manager functionalities (e.g. position list and stage control) to support freely defined primary scan positions for this platform (currently only supported with Leica LAS AF/X), and the possible control of hardware triggers. Developing custom image analysis scripts to tackle new applications in ImageJ macro language should be relatively straightforward but we are aware that this might still be a hurdle for some inexperienced users. Also, the range of possible applications might be limited by the complexity of the underlying analysis. As such, we plan to tighten the links with emerging ImageJ deep learning frameworks and model zoos, for instance as a new AutoScanJ module meant to train deep learning networks from annotated primary scans, and then make using this classifier in an AutoScanJ target detection function straightforward. Finally, while we expect that most microscopy modalities should be supported by the combination of Micro-Manager and one of the main commercial microscope manufacturers, we would like to support more commercial systems and simplify bridging AutoScanJ to open microscopy projects and custom systems (e.g. Labview based). To facilitate this, we plan to provision pre-packaged bridges for a number of microscope manufacturers. If these acquisition platforms do not enable network message triggered events in the acquisition protocols, we will consider Daemon like applications running in the background and bridging AutoScanJ network messages to their respective external communication protocols.

## Data Availability

The datasets presented in this study can be found in online repositories. The names of the repository/repositories and accession numbers can be found in the article/Supplementary Material.

## Footnotes

1 Driven from LAS AF or LAS X (from 1.x to 3.x), with both Matrix and Computer Assisted Microscopy modules installed.
