# Supplementary material for: AutoScanJ: A Suite of ImageJ Scripts for Intelligent Microscopy
Source: Front Bioinform. 2021 Mar 18;1:627626. doi: 10.3389/fbinf.2021.627626 (PMC9581036; doi:10.3389/fbinf.2021.627626)
Supplement: Supplementary file 1 [file datasheet1.pdf]

Supplementary Data for

AutoScanJ: A Suite of ImageJ Scripts

for Intelligent Microscopy

Sébastien Tosi[1], Anna Lladó[1], Lídia Bardia[1], Elena Rebollo[2], Anna Godo[3], Petra Stockinger[4], Julien Colombelli[1]

[1]Institute for Research in Biomedicine, IRB Barcelona, Barcelona Institute of Science and Technology, BIST, Barcelona, Spain
[2]Molecular Imaging Platform, Molecular Biology institute of Barcelona IBMB-CSIC, Barcelona, Spain
[3]Genetics of Male Fertility Group, Cell Biology Unit, Faculty of Biosciences, Autonomous University of Barcelona, Bellaterra, Cerdanyola del Vallès, Barcelona, Spain
[4]Center for Genomic Regulation, CRG; Barcelona Institute of Science and Technology, BIST, Barcelona, Spain

Table of Contents

S0 - Software and Hardware Components 2
S1 - Videos 7
S2 - Sample Data 9
S3 - Image Analysis Workflows 10
S4 - AutoScanJ Documentation 13
S5 - Writing a Custom Target Detection Function 13
S6 - Sample Preparation and Imaging Methods 17
S7 - Imaging Instruments 20
S8 - Open Source Intelligent Microscopy Software 21
References 23

S0 - Software and Hardware Components

S0.0 Hardware requirements and considerations

The acquisition software (Leica LAS AF/X or Micro-Manager should be able to control the microscope XY stage, objective Z drive and switch emission/detection fluorescent filters. The objective turret does not necessarily need to be motorized when using the same objective for both primary and secondary scans (or for fixed experiment since the switch can be performed manually between scans). <u>An objective switch for live experiments should only be performed if both objectives are dry</u>.

All the applications described in the article were performed with microscopes not equipped with hardware autofocus. To adjust the focus we only relied on the software focus map set prior to the primary scan (from Leica LAS AF/X Matrix module or AutoScanJ Micro-Manager script) and the acquisition software fine autofocus (optionally run prior to acquiring each secondary target and starting from the interpolated position estimated from the focus map). When using Micro-Manager, an option is provided to set the focus manually at the first AF call. An offset is computed between this manual focus setting and the focus map and it is maintained to set the starting Z positions of the subsequent AF calls. This might be useful if the focus map estimate is too coarse, or when switching between non parfocal objective lenses between primary and secondary scan.

When using a high magnification objective lens during the secondary scan, it is often more favorable to use an intermediate magnification objective lens during the primary scan to tile a large area of the sample than using a low magnification objective and very few tiles. This is because the positions of the secondary targets are estimated from the center of the images <u>assuming no residual camera (or confocal scanner tilt) respect to the stage coordinates system</u>. The primary scan can then be accelerated by relying on binning and relatively lower exposure time (or lower confocal zoom, larger pinhole and higher scan speed).

S0.1 Interactions between hardware and software components

AutoScanJ implements a client-server architecture enabling to perform demanding target detection image analysis on a dedicated workstation (**Figure S0.1, IP-WS**). This can for instance be useful to handle the analysis of especially large primary scans, but it is not strictly required otherwise since there is no risk that target detection interferes with image acquisition (it is always performed in between primary/secondary scans). When using an **IP-WS**, this machine must be visible from the microscope workstation (**Fig. S0.1, Mic-WS**) and the IP of this machine must be set from the dialog box of AutoScanJ macro to enable communication between both workstations. Image files are simply shared from a network folder (that should be visible by both machines and set as the image exportation folder). When using the **Mic-WS** to perform target detection, the IP is set to **localhost** (127.0.0.0) and the images are exported to a local folder.

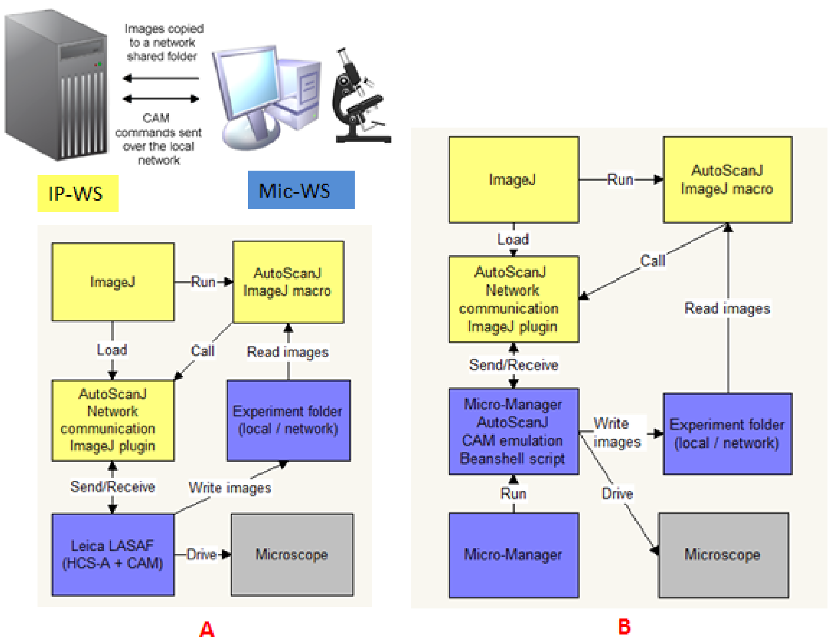

Figure S0.1 Interaction between the different hardware and software components used by AutoScanJ to perform intelligent microscopy. The color of the boxes represents the hardware on which each software component is run: Image Processing workstation **IP-WS** (yellow), Microscope Workstation **Mic-WS** (blue)

Irrespective of the client-server configuration and of the acquisition software used to drive the microscope (Micro-Manager, **Fig. S0.1B** or Leica LASAF, **Fig. S0.1A**), AutoScanJ ImageJ macro sends CAM network commands to control the microscope. The network communication is handled on the ImageJ side (client) by calling a custom network communication plugin and by Leica LAS AF/X or Micro-Manager (server). To enable this, when using Micro-Manager, a custom AutoScanJ script (Beanshell) has to be loaded (**Fig. S0.1B**). This script emulates a Leica CAM server and it orchestrates the operations performed during the primary and the secondary scans (**Fig. S0.4**).

S0.2 Sequence of operations and required user configuration

The sequence of user configuration steps and the operations automated by AutoScanJ ImageJ macro are depicted in **Fig S0-2** for a fixed sample tiled experiment.

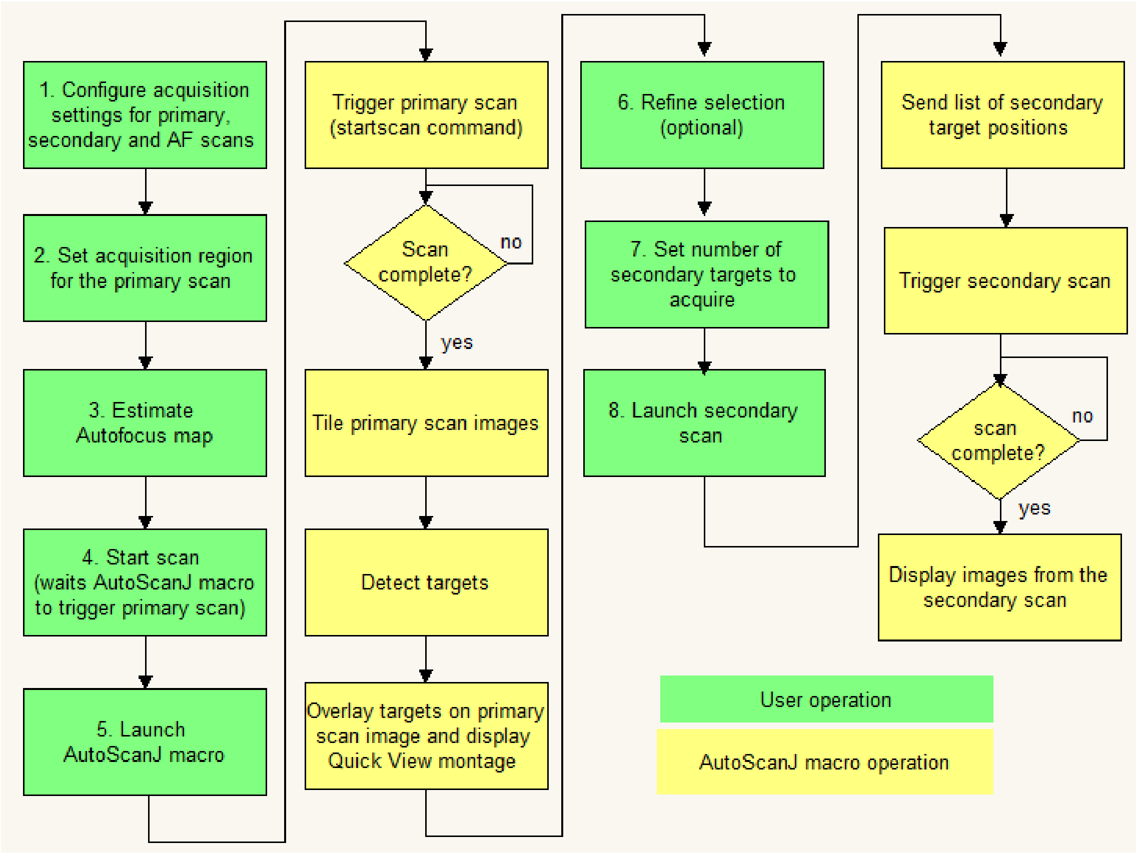

Figure S0.2 -Sequence of operations performed by the user (green) and AutoScanJ macro (yellow) when using AutoScanJ with fixed sample and tiling mode

First, the primary and secondary scans have to be manually configured. This consists in 1) setting an image exportation folder and the acquisition parameters (channels, objective, binning / zoom, Z slice spacing ...) associated to the primary, secondary and fine autofocus scans, and 2) setting the acquisition region of the primary scan (number of fields of views, positions). This procedure is described in detail in AutoScanJ documentation (**Supplementary Section S4**).

Next, an autofocus map is estimated (3). This is achieved either from the user interface of Leica LAS AF/X Matrix module (**Fig. S0.3A, left**), or from the dialog box of AutoScanJ Micro-manager script (**Fig. S0.3A, right**). When using Leica LAS AF, the focus map is estimated from user defined control points spread across the primary scan region while for Micro-Manager, the focus map is more simply estimated from user adjusted focus at the four corners of the primary scan tile grid (**Fig. S0.3B**).

Next, the user sends the acquisition software in waiting mode (4) until a CAM command triggers the primary scan. For this, the user needs to launch AutoScanJ macro (5) and configures some basic information from its dialog box (**Mic-WS** IP, exportation folder matching the previous folder and target detection function).

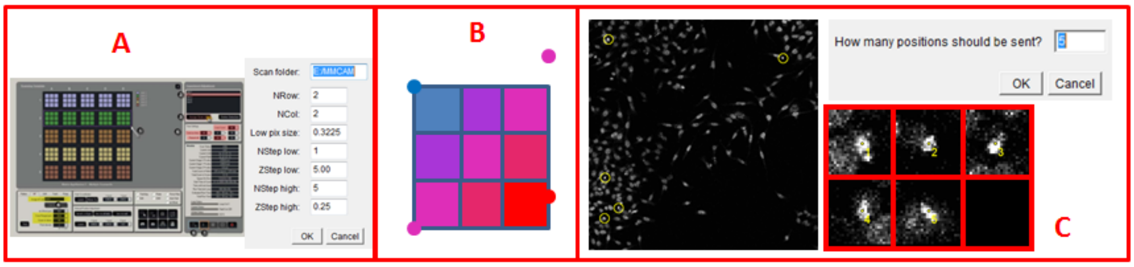

Figure S0.3 Main manual configuration steps when using AutoScanJ with fixed sample and tiling mode. (A) Leica LAS AF/X Matrix module UI and AutoScanJ Micro-Manager script to configure primary and secondary scans **(B)** Graphical representation of the Autofocus map estimated by Micro-Manager AutoScanJ script from the user adjusted focus of the 4 fields of view cornering the primary scan. **(C)** Primary scan map with targets detected by the target detection function. The user can edit the selection from the montage or the Quick View montage, and can then acquire all or only the first **N** targets during the secondary scan.

AutoScanJ macro triggers the primary scan and it waits for the primary scan to complete (a CAM command is sent by **Mic-WS** upon a scan completion). Next, the macro loads the images from the primary scan, performs their maximum intensity projection, and tiles the projected images before calling the target detection function. The user can then refine the secondary target selection (6) by deselecting erroneous detections from the Quick View montage (**Fig. S0.3, C**). It is also possible to (7) limit the number of targets to acquire during the secondary scan from a dialog box (**Fig. S0.3C**) before (8) launching the secondary scan.

During the secondary scan, the targets are sequentially wandered by the acquisition software (**Fig. S0.4**). A fine autofocus can optionally be run prior to acquiring each secondary target (starting from the position interpolated from the focus map). When using Micro-Manager, an option is provided to set the focus manually at the first AF call. An offset is computed between this manual focus setting and the focus map and it is maintained to set the starting Z positions of the subsequent AF calls. This might be useful if the focus map estimate is too coarse, or when switching between non parfocal objective lenses between primary and secondary scan.

It is also possible to repeat the same secondary scan multiple times, that is to reacquire the same targets multiple times by manually re-launching the macro with this option. This can be useful to perform several rounds of washing and staining of the same sample, provided it can be repositioned reproducibly on the stage. The stage itself can be positioned at the same absolute coordinates prior to each secondary scan from the acquisition software.

The operations for live sample experiments are very similar (Main Text, **Fig. 1**), except that no user interaction is performed after target detection and that the primary scan is automatically triggered periodically and that. For block experiments, the target refinement step can also be skipped so that a large number of blocks can be processed without user interaction; the targets detected in each block are then sequentially accumulated to an overall target detection list prior to running the secondary scan (Main Text, **Fig. 1**).

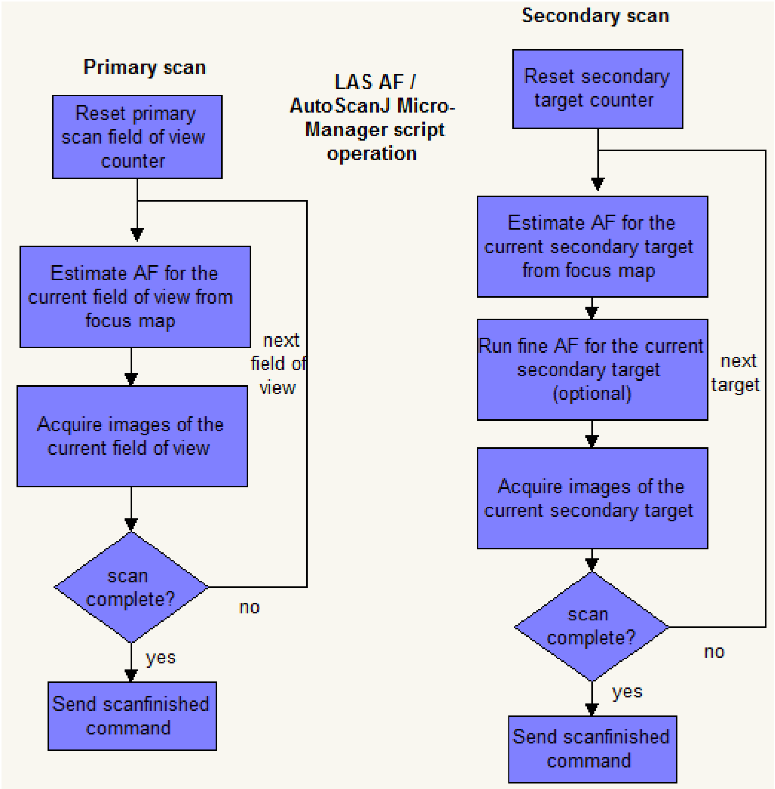

Figure S0.4 Sequence of operations performed by the acquisition software (LAS AF/X or Micro-Manager AutoScanJ script) during the primary scan (left) and secondary scan (right)

S0.3 Subset of Leica CAM commands used by AutoScanJ to synchronize operations

AutoScanJ macro and the acquisition software (Leica LAS AF or Micro-Manager) exchanges CAM network commands [Leica 20] to synchronize operations and send secondary scan target positions. This is achieved with a subset of Leica CAM commands that is reported in **Table S0**.

**Start primary scan**	/cmd:**startscan**
**Delete target position list**	/cmd:**deletelist**
**Append position to target list**	/cmd:**add** /tar:camlist /exp:Job high /ext:none /slide:0 /wellx:1 /welly:1 /fieldx:1 /fieldy:1 /dxpos:-63 /dypos:-63
**Start secondary scan**	/cmd:**startcamscan** /runtime:9999 /repeattime:9999
**Scan finished**	/cmd:**scanfinished**

Table S0 Subset of Leica CAM commands used to synchronize operations and send information between AutoScanJ macro and the acquisition software

S1 - Videos

S1-V1 LLC-PK cells primary scan (MIP time-lapse)

Primary scan (mCherry-α-tubulin) demonstrating mitosis onset detection (white circles) of LLC-PK live cells. Maximum intensity projection of the original images. The time-lapse is frozen during 3 frames after each mitosis detection. Time scale shows hours and minutes.

LLC-PK_primary_mitosis_detection.mp4

S1-V2 LLC-PK cells secondary scan (MIP time-lapse)

High-resolution time-lapses acquired during the secondary scan for mitosis detected in video **S1-V1** (α-tubulin: green, Centrin: red). Maximum intensity projection of the original images. Time scale shows hours and minutes.

LLC-PK_secondary_detected_mitosis.mp4

S1-V3 Neuroblasts primary scan (MIP time-lapse)

Primary scan (His2Av-eGFP) demonstrating neuroblast mitosis detection (white circles). 12 independent blocks holding a neuroblast are monitored in parallel, the numbers in each block are related to the probability of a neuroblast not dividing in the current frame (see **S3-2**). Maximum intensity projection of the original images. The time-lapse is frozen during the time of the secondary scan triggered after each mitosis detection. Time scale shows seconds.

Neuroblast_primary_mitosis_detection.mp4

S1-V4 Neuroblasts secondary scan (MIP time-lapse)

Four high-resolution time-lapses (His2Av) acquired during the secondary scan for mitosis detected in video **S1-V3** . Maximum intensity projection of the original images. Time scale shows seconds.

Neuroblast_secondary_detected_mitosis.mp4

S1-V5 Neuroblasts secondary scan (3D MIP volume rendering time-lapse)

3D rendering of one of the four time-lapses from Video **S1-SV4**. The video is played forward and then backward to better understand the ongoing process.

Neuroblast_secondary_3D.mp4

S1-V6 Neuroblasts primary scan target detection pipeline

This video illustrates the live detection of neuroblasts mitosis by exemplifying the image analysis workflow detailed in **S3-2**.

Neuroblast_primary_sample_IA.mp4

S1-V7 Abnormal human spermatozoa (3D MIP volume rendering and spots segmentation)

3D rendering of one of the abnormal nuclei shown in Supplementary Figure S3-4. This phenotype presents a disomy (2 copies of chromosome 18, shown in cyan) and chromosome X (in yellow) and Y (in magenta). The three FISH and the nucleus (DAPI signal, shown in grey) are segmented in the last frames of the video.

FISH_secondary_abnormal_nucleus.mp4

S2 - Sample Data

Raw images from the primary scans of some of the experiments presented in the main text can be downloaded from https://bit.ly/3obNrBT, and possibly used to test the macro in *offline* mode (that is with no microscope connection). Usage instructions can be found here: https://github.com/SebastienTs/AutoScanJ.

S2-D1 Imaging mitotic cells at high resolution

FixedSample_Tiling_HeLa_Metaphase.zip

Primary scan images from the application *Imaging mitotic cells at high resolution*. Compatible with AutoScanJ sample workflow ***_Metaphase_detector*** (Tiled/Fixed).

S2-D2 Studying isolated cells on Fibronectin micro-patterned surfaces

FixedSample_Blocks_Cytoo_Isolated_Cells.zip

Primary scan images from the application *Acquiring isolated cells seeded on Fibronectin micro-patterned surfaces.* Compatible with AutoScanJ sample workflow ***_Cytoo_Isolated_Nuclei_SP5*** (Block/Fixed).

S2-D3 Fluorescent Kidney slides glomeruli detection

FixedSample_Tiling_Kidney_Slice_Glomerulus.zip

Primary scan images demonstrating the detection of textured objects in fluorescent Kidney slides (GFP channel) Invitrogen FluoCells™ Prepared Slide #3. This sample is only used to test the system from primary scan target detection from AutoScanJ sample workflow ***_Glomerulus_detector*** (Tiled/Fixed). See **S6-M6** for the imaging settings used to acquire these primary scan images (and that should be used to test AutoScanJ with this sample on your system).

S2-D4 Acquiring mitosis events from their onset

LiveSample_Tiling_LCPK_Cells_Mitosis_Onset.zip

Primary scan images from the application *Acquiring mitosis events from their onset***.** Compatible with AutoScanJ sample workflow ***_Mitosis_Microtubulin*** (Tiled/Live).

S3 - Image Analysis Workflows

In this section we provide further information on the target detection image analysis workflows which were not fully detailed in the article.

S3.1 Pattern and single nuclei detection (Cytoo application)

The analysis of a Cytoo block is performed by first identifying valid patterns from the primary scan images of the Fibronectin fluorescence channel (**Fig. S3.1A**). Fibronectin patterns are detected by following steps **a1** to **a3** (**Fig. S3.1)**. To avoid spurious detection, only patterns aligned both vertically and horizontally (within a given tolerance) with at least **N** other patterns are considered valid. A fixed size square region centered on each valid pattern is then analyzed in the DAPI channel (squares in **Fig. S3.1B**). The sequence of operations to count the number of nuclei in these regions is illustrated in steps **b1** to **b4** (**Fig. S3.1**)**.** Only connected particles above a minimum area are counted as valid nuclei and patterns holding single cells (bright squares in **Fig. S3.1B**) are acquired during the secondary scan (**Fig S3.1C**).

S3.2 Neuroblast mitosis detection

To detect the onset of neuroblast mitosis, the fields of view of the blocks are initially centered on neuroblasts and are subsequently periodically acquired and analyzed sequentially. For this, the image of a block at frame $\boldsymbol{t}$ (**Fig. S3.2, a1**) is processed by a Gaussian blur filter followed by global thresholding and binary watersheding (**Fig. S3.2, a2**).

The area $\boldsymbol{A_i(t)}$ and mean intensity $\boldsymbol{M_i(t)}$ of each connected particle $\boldsymbol{i}$ found in the resulting mask are estimated. A fitness function $\boldsymbol{F_i(t)}$ is then computed from these measurements (**Fig. S3.2,right**). The parameters $\boldsymbol{A_0}$ and $\boldsymbol{M_0}$ of this function are calibrated to the characteristic area and average intensity observed in non-dividing neuroblasts so that the fitness function takes values close to 1 if the particle $\boldsymbol{i}$ resembles a non-dividing neuroblast (and relatively lower values otherwise). The overall fitness is computed as the maximum over all the fitness function $\boldsymbol{F_i(t)}$ and it is hence only close to 1 if at least one particle resembles a non-dividing neuroblast in the image. To make the detection sufficiently robust, a neuroblast mitosis onset event is only triggered if the fitness function falls from a value above the monitoring threshold (set around 0.85) below the detection threshold (set around 0.775) and if the fall from the previous time frame is greater than the drop threshold (set around 0.15). See the video **S1-V7** for a live illustration of mitosis detection and real-time computation of the fitness function.

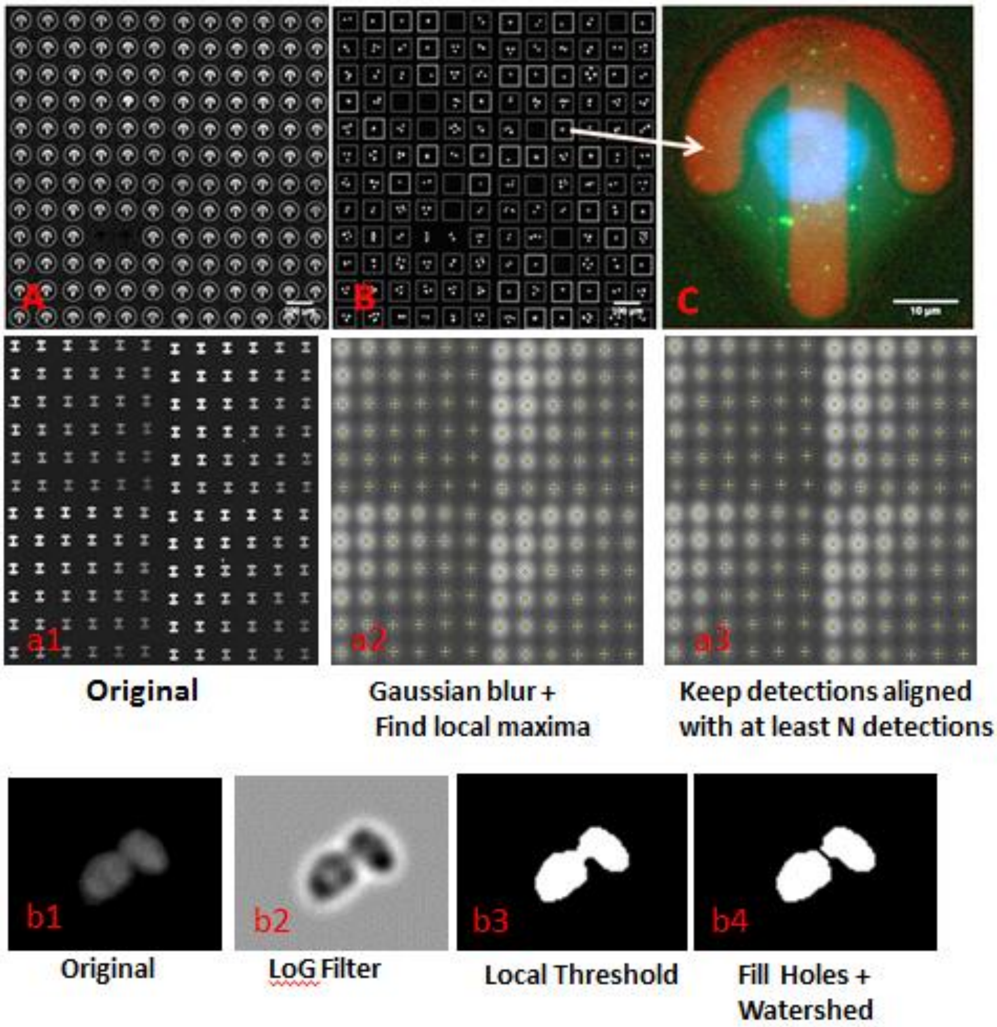

Figure S3.1 Steps performed to identify valid Cytoo patterns (**a1** to **a3**, valid patterns circled in **A**), and detect patterns with only a single nucleus (**b1** to **b4**, single nuclei highlighted by bright squares in **B**). Only the single cells sitting on a valid pattern are acquired during the secondary scan (**C**).

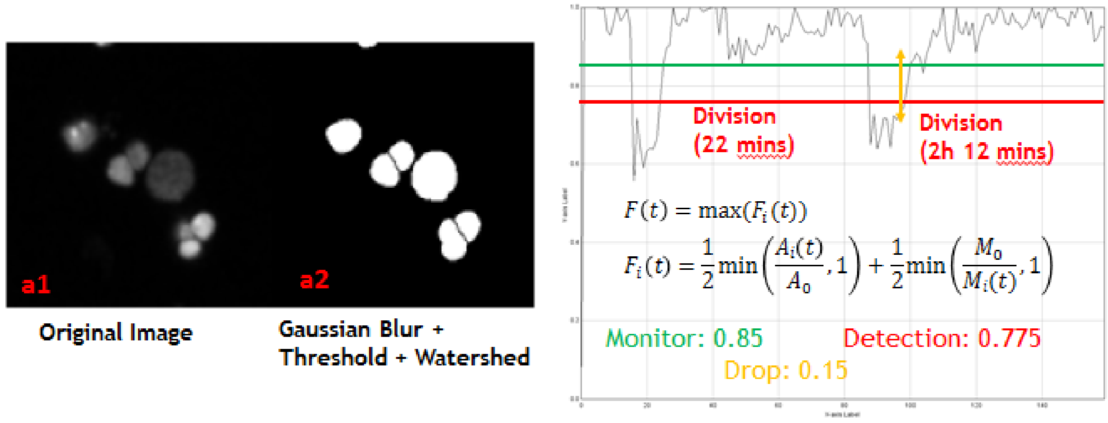

Figure S3.2 Sample image of a non-dividing neuroblast and surrounding daughter cells a1: original image. **a2**: Resulting binary mask and connected particles analysis. **Right**: Time plot of the fitness function ***F(t)*** for a field of view in which a neuroblast undergoes two divisions. The divisions are only detected when the fitness function quickly falls from monitor to detection threshold.

S3.3 FISH Application

In the FISH application, human spermatozoa exhibiting abnormal genotypes were studied using FISH probes. An example of high 3D resolution images of nuclei showing a set of different phenotypes taken during AutoScanJ secondary scan is shown below (Maximum intensity projections in Figure **S3.3**) and a 3D MIP volume rendering and spots segmentation in Supplementary video **S1-V6**.

A standalone ImageJ macro implementing a similar target detection image analysis workflow than the one used to segment and classify nuclei for the FISH application can be downloaded from IRB Barcelona ADMCF webpage here. The code is provided together with a sample primary scan image montage acquired during the project.

A complete tutorial describing a very similar image analysis workflow and associated programming concepts is available in the chapter 5 of our book on BioImage Analysis: https://analyticalscience.wiley.com/do/10.1002/was.00050003.

The original AutoScanJ analysis function that was used for the project can be downloaded here bit.ly/3nzOcmZ. It however requires an experimental version of AutoScanJ **Fixed_Tiling.ijm** macro (provided at think as well) that can handle an unlimited number of channels during the primary scan (no RGB montaging).

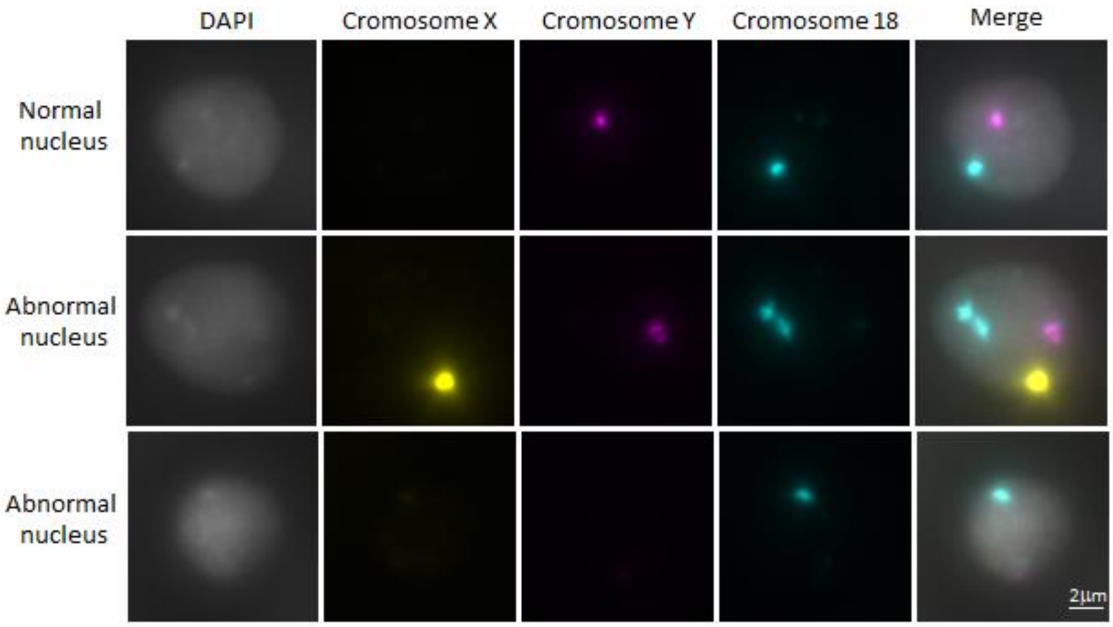

Figure S3.3 Human sperm cells acquired during AutoScanJ secondary scan. Maximum intensity projections of human spermatozoa are shown. In normal spermatozoa either no chromosome X or Y signal is expected. The abnormal nucleus from the middle row presents a disomy (2 copies of chromosome 18) and the nucleus from the last row a nullisomy (no chromosome X nor Y).

S4 - AutoScanJ Documentation

AutoScanJ Micro-Manager documentation: **https://bit.ly/2SyWcHS**

AutoScanJ Leica LAS AF documentation: **https://bit.ly/2GEhu4a**

S5 - Writing a Custom Target Detection Function

A simple way to write and debug a target detection function for a new application is to first acquire some illustrative images of the sample by running a primary scan without secondary scan, and then testing the target detection with the montage of the primary scan images exported by AutoScanJ (exportation folder). Once the detection is satisfying, the macro can be packaged as an AutoScanJ detection function and tested by running AutoScanJ offline (without communication to the microscope) on the same primary scan images first, and then by performing a complete experiment including primary and secondary scans.

Writing a fixed experiment event detection function

1. **Acquiring primary scan sample images**

Use the version of AutoScanJ corresponding to the application you plan (e.g. **FixedSample_Tiling.ijm**) and perform a primary scan without target detection function (set **Automatic pre-analysis** to ***None*** from the dialog box). The images of the primary scan are exported to disk and the acquisition can then be exited without acquiring secondary scan images (no hand selection on the montage or set the number of secondary targets to acquire to 0).

2. **Writing the image analysis workflow as an ImageJ macro**

The image analysis workflow should first be tested on the primary scan map (**Montage.tif**) image exported by AutoScanJ (in the exportation folder). If the montage is large, it is advised to first start with a cropped out region to make testing and debugging faster. Note that the montage is a tiled maximum intensity projection of the original images but, depending on the number of channels configured for the primary scan, it can be either grayscale (one channel) or RGB (up to three channels). To write the ImageJ macro implementing the target detection image analysis workflow, open ImageJ script editor (File > New > Script) and set the language to ImageJ macro (Language > ImageJ Macro). Writing the workflow requires a basic knowledge of ImageJ macro language. We do not cover this here, but a good starting point is to use the macro recorder and the webpage: https://imagej.nih.gov/ij/developer/macro/macros.html. A complete introduction to ImageJ macro language in the context of bioimage analysis is provided in this book: https://analyticalscience.wiley.com/do/10.1002/was.00050003.

3. **Integrating the code to a new target function**

The macro code must now be integrated to an ImageJ macro function and appended to the corresponding AutoScanJ file (e.g. **AnalysisFunctions_Fixed_Tiling.ijm**). This way, AutoScanJ can parse the file and automatically register the corresponding target detection functions so as to make them available from the main dialog box. Importantly, when the target detection function is called, the only open image is the primary scan map. AutoScanJ expects the function to exit with only this same image open and with a multi-point selection locating the positions of the detected targets. The function name must start with an underscore. This is required so that AutoScanJ registers the function correctly when parsing the file. A generic bright object detector is provided in the code section below.

```
function _BrightObjects_detector(ImagesSize)
{
	// Initialization
	OriginalID = getImageID();
	run("Duplicate...", "title=FilteredImage");

	// Processing
	run("Median...", "radius=2");
	setAutoThreshold("Otsu dark");
	run("Analyze Particles...", "size=0-Infinity circularity=0.00-1.00 show=Nothing display clear add include");

	// Close intermediate images
	selectImage("FilteredImage");
	close();

	// Create multi-point selection at detected targets locations
	selectImage(OriginalID);
	for(i=0;i<nResults;i++)
	{
		makePoint(getResult("X",i),getResult("Y",i));
		setKeyDown("shift");
	}
	setTool("multipoint");
}
```

In the initialization sequence the original active image (primary scan map) is duplicated. Then, the functional section of the code (in red) performs a sequence of steps to detect bright objects of a specific shape in the image and store the coordinates of the centroids of the detected targets in ImageJ results table. Finally, the last section reads this information and creates a multi-point selection at the corresponding coordinates on the original image. This image should be the only one left open.

4. Testing the target function

AutoScanJ can now be run and the new target function should be available from the **Automatic pre-analysis** drop-down menu. To test the function without re-acquiring new primary scan images, run AutoScanJ without ticking "**Perform primary scan**" and "**Send CAM scripts**" and select the same exportation folder that you used to acquire the primary scan images in Step 1. Since the scripts are not sent to the microscope acquisition software, this can be conveniently performed on a machine not necessarily connected to the microscope. Check the detected targets from the primary scan map or from the Quick View montage. If the detection is satisfactory you can now run a complete experiment!

Guidelines when writing a detection function

- Depending on the complexity of the detection task at hand (and the reproducibility of the sample preparation and imaging), several iterations of acquisition of primary images and troubleshooting / optimization of the target detection function may have to be performed until the accuracy is deemed sufficient for the application.

- For fixed samples, since the target selection can be refined by the user from the Quick View montage, the detection function should rather be permissive (a fraction of false positive) than too conservative (undetected targets). Obviously, the highest detection accuracy is always desirable to relieve user burden.

- For fixed samples, the points of the multi-point selection should ideally be ordered based on the spatial locations of the targets. This way, a target detected multiple times can be easily eliminated from the Quick View montage.

- For live experiments, since the microscope acquires secondary scan targets autonomously (without user refinement), the target detection should rather not be too permissive since the throughput of usable experiment data will drop if too many false positive events are detected. Still, especially when the events of interest are very scarce, the detection function should be as accurate as possible.

Writing a live experiment event detection function

For live experiments the primary scan maps hold a buffer of the last three time frames of the experiments to enable events detection. When the target detection starts it should hence expect a 3 time frame ImageJ hyperstack to be open. AutoScanJ still expects that the function exits with this original image active, but in this case only a single point selection should be set at the location of a detected event. In case multiple events are detected in this time frame, the detection function should only report one of them (e.g. the most probable).

The easiest way to debug detection functions for live experiments is to use AutoScanJ directly in offline mode (Step 3) with the images of a pre-acquired uninterrupted primary scan (no target detection). The procedure and usage with the sample data from section **S2** is fully detailed from: https://github.com/SebastienTs/AutoScanJ, but to prepare the data one should basically:

- Create an empty folder before each new run
- Set the variable ***OfflineFilesPath*** in AutoScanJ macro to the path of the folder with the images of the uninterrupted primary scan
- Run the ImageJ macro and select the empty folder as experiment folder
- Un-tick ***Send CAM scripts*** so that the macro does not attempt to control the microscope

S6 - Sample Preparation and Imaging Methods

S6-M1 HeLa cells mitosis

HeLa cells were seeded on coverslips. After paraformaldehyde fixation, immunostaining was done as described in (Sdelci et al 2012). Briefly, tubulin was labeled using mouse anti-β-tubulin antibody (sigma), DNA was labeled with DAPI and centrosomes were labeled with rabbit anti-pericentrin (donated by CRG). Secondary antibodies used were Alexa Fluor 555 goat anti-mouse IgG and Alexa Fluor 647 goat anti-rabbit IgG (Invitrogen).

Primary scan: Leica TCS SP5 inverted confocal fluorescence microscope (see section S7.1). DAPI channel. 10x10 images tiled map (256x256 pixel each), objective: 63x Oil (NA=1.4), Zoom: 1, Pinhole: 3 Airy units. Pixel size: 965nm.

Secondary scan: Leica TCS SP5 inverted confocal fluorescence microscope (see section S7.1). DAPI, Alexa Fluor 555 and Alexa Fluor 647 channels. 1024x1024 pixels image, objective: 63x Oil (NA=1.4), Zoom: 3, 65 Z-slices (125 nm spacing), Pinhole: 1 Airy unit. Pixel size: 80 nm.

S6-M2 Human sperm cells sample preparation for FISH

Semen samples were processed for FISH analysis according to (Sarrate and Anton 2009). In brief, this includes sperm fixation in methanol:acetic acid (3:1), manual spreading on slides, sperm chromatin denaturalization in 5mM DTT (dithiothreitol), DNA denaturalization in formamide (70%) and probe hybridization. A sequential FISH protocol was conducted as described in (Godo et al. 2013).

In the first hybridization for the primary scan, a combination of centromeric probes for chromosomes 18, X and Y were used (CEP 18, D18Z1, Spectrum Aqua; CEP X, DXZ1, Spectrum Green; CEP Y, DYZ3, Spectrum Orange [AneuVysion Multicolor DNA Probe Kit, Abbott Molecular Inc., Des Plaines, IL, USA]). In the second hybridization round (for the secondary resolution scan only), a combination of centromeric probes for chromosomes 6, 8 and 9 were used (CEP 6, Spectrum Green; CEP 8, Spectrum Aqua; CEP 9, Spectrum Orange [Abbott Molecular Inc.]).

Primary scan: Leica DMI 6000B inverted widefield fluorescence microscope (see section S7.2). DAPI channel. 6x6 fields of view, objective: 20x (NA=0.7), Camera binning: 2, 3 Z slices (3 um spacing). Pixel size: 645 nm.

Secondary scan: Leica DMI 6000B inverted widefield fluorescence microscope (see section S7.2). DAPI, Spectrum Aqua, Spectrum Green and Spectrum Orange channels. Objective: 63x Oil (NA=1.3), Camera binning: 1, 59 Z slices (0.3 um spacing). Pixel size: 102 nm.

S6-M3 Micropatterned surfaces and HeLa cells

HeLa cells stably expressing GFP-LC3 (LC3 corresponds to protein-microtubule-associated-protein 1 light chain 3, an autophagosome protein) were seeded at very low dilution on CYTOO starter chips labeled with Fibronectin-Alexa 647 (CYTOO). After a few hours, they were paraformaldehyde fixed (Electron Microscopy Sciences) and stained with DAPI 1306 (ThermoFisher Scientific). ProLong Gold antifade reagent (ThermoFisher Scientific) was used for mounting the chips on slides.

Primary scan: Leica TCS SP5 inverted confocal fluorescence microscope (see section S7.1). 2x2 tiles (1024x1024 pixels each) per block. Objective: 20x (NA=0.7), Zoom: 1, Pinhole: 3 Airy units. Pixel size: 758 nm.

Secondary scan: Leica TCS SP5 inverted confocal fluorescence microscope (see section S7.1). 1024 x 1024 pixels. Objective: 20x (NA=0.7), Zoom: 3, Pinhole: 1 Airy unit, 25 Z slices (0.5 µm spacing). Pixel size: 252 nm.

S6-M4 LLC-PK cells culture

The mCherry-α-tubulin plasmid was obtained from Addgene (Addgene 21043). The Centrin-2-GFP plasmid was a generous gift from Tim Stearns (Stanford University, USA). LLC-PK1 cells were grown in DMEM containing 10% fetal calf serum. To generate a LLC-PK cell line stably expressing Centrin-GFP and mCherry-α-tubulin, cells were transfected with corresponding plasmids and selected in the presence of 0.4 µg/ml geneticin. Resistant clones were isolated and tested for expression of the tagged proteins.

Primary scan: Leica TCS SP5 inverted confocal fluorescence microscope (see section S7.1). mCherry channel. 3x2 tiles (512x512 pixels each). Objective: 63x Oil (NA=1.4), Zoom x1, Pinhole 3 Airy units, 3 Z slices (2 µm spacing), repetition time: 2 minutes. Pixel size: 482 nm. Experiment length: 16h.

Secondary scan: Leica TCS SP5 inverted confocal fluorescence microscope (see section S7.1). mCherry and GFP channel. 1024x1024 pixels. Objective: 63x Oil (NA=1.4), Zoom x3, Pinhole 1 Airy unit, 7 Z slices (1 µm spacing), repetition time: 5 minutes. Pixel size: 80 nm. Time-lapses length: 1h.

S6-M5 Neuroblast primary culture

Drosophila brains of 3rd instar larvae expressing His2Av-eGFP (Histone H2A, Bloomington 24163) were dissected in cold collagenase buffer (800 mg NaCl, 20 mg KCl, and 5 mg NaH_2PO_4, 100 mg $NaHCO_3$ and 100 mg Glucose in 100 ml ddH_2O) and incubated in 0.2 mg/ml collagenase (Sigma) for 20 min at room temperature (Feiguin, Llamazares and González, 1997). Brains were rinsed in collagenase buffer and manually dissociated in Schneider's medium complemented with 5% FCS, glucose (1mg/ml), 5% fly serum (Milner lab) and insulin (5 µg/ml) through pipetting.

Imaging conditions

Neuroblast cells were plated on poly-L-lysine-coated 35 mm glass bottom Fluorodish culture plates (WPI) and allowed to settle for 30 mins at room temperature. Live imaging of primary brain cultures was performed at room temperature (22-25°C) using a Leica TCS SP5 (inverted) confocal microscope with a 63x/1.4 NA oil objective using a 488 Argon laser.

Primary scan: Leica SP5 inverted confocal fluorescence microscope (see section S7.1). eGFP channel. 12 blocks (256x256 pixels each), 63x Oil (NA=1.4), Zoom x6, Pinhole 3 Airy units, 5 Z slices (2 µm spacing), repetition time: 1.5 minutes. Pixel size: 160 nm. Experiment length: 4h.

Secondary scan: Leica SP5 inverted confocal fluorescence microscope (see section S7.1). eGFP channel. 512x512 pixels, 63x Oil (NA=1.4), Zoom x6, Pinhole 1 Airy unit, 21 Z slices (1 µm spacing), repetition time: 30 s. Pixel size: 80 nm. Time-lapses length: 25 minutes.

S6-M6 Fluorescent Kidney slides glomeruli detection

No sample preparation is involved since this is a commercial slide. We report below the primary scan imaging conditions of the sample dataset **S2-D3** for which no secondary are provided (they are not relevant as this sample is only used to test the system from primary scan target detection). When using the test sample to perform a test experiment on a different microscope similar imaging conditions should be used (the sample has also been tested with widefield microscopes).

Primary scan: Leica SP5 inverted confocal fluorescence microscope (see section S7.1). GFP channel. 3x2 tiles (1024x1024 pixels each), 10x Dry (NA=0.5), Zoom x1, Pinhole 2 Airy units, 1 Z slice. Pixel size: 1.32 um.

S7 - Imaging Instruments

S7.1 Confocal microscope

Leica TCS SP5 inverted confocal microscope with high-efficiency spectral PMT detection (five simultaneous channels) and AOBS module (Acousto Optical Beam Splitter). The laser lines available are 405 nm blue diode laser, argon laser (458, 476, 488, 496, 514 nm), 561 nm DPSS laser and helium-neon lasers (594, 633 nm). The microscope is equipped with a Märzhäuser motorized stage, C02 and temperature controller incubator (Life Imaging Services), and controlled by Leica LAS AF software with licenses for both Matrix and Computer Aided Microscopy (CAM) modules.

S7.2 Widefield microscope

Leica DMI6000B inverted, fully automated microscope, equipped with a dry 20x (NA=0.7) and an oil immersion 63x (NA=1.3) apochromat objectives, a digital CCD camera ORCA-R2, a scanning stage (0.05 m resolution) and a Metal Halide Bulb (HPX120) as illumination source. Two full filter sets are specifically built in for the green (BP482/18; 495; BP520/28) and orange (BP546/10; 560;BP585/40) spectra respectively. Additionally, the system contains a doubleband Pinkel filter set for the cyan (-; 453; BP467/23) and yellow (-; 518;BP535/30) spectra, associated to an external ultrafast excitation wheel containing respectively the BP430 and BP500 filters, among others. All the components can be controlled from Micro-Manager, including the external filter wheel, for which a Dynamic Link Library (DLL) file was purposely developed by the Micro-Manager team during this project.

S8 - Open Source Intelligent Microscopy Software

We report an indicative list (Table S1) of open source software performing intelligent microscopy or a degree of online feedback microscopy together with related important features such as the acquisition software they are compatible with, the image analysis software they rely on, the modalities supported during the primary and secondary scans (2D/3D, Fixed/Live) and the main applications demonstrated in published scientific articles. This list does not pretend to be exhaustive as a large number of projects are based on custom hardware and software, but we tried to reflect most projects for which a substantial effort has been made to document and promote reusability. For each software, we tried to reflect the **R**eusability (simplicity, as of today, to use the system for the same application, non-commercial software), **F**lexibility (range of supported microscopes, adaptability to slightly different samples, possibility to offload image analysis) and **V**ersatility (range of acquisition modalities, extendibility to new acquisition modalities, applications demonstrated) with a qualitative rating (from ++ to -). To be fair, versatility is not rated for software explicitly developed for a specific application.

Software Subjective R / F / V (NR: not rated)	Acquisition software	Image Analysis	Primary scan Imaging	Secondary scan Imaging	Demonstrated Applications
**Primary scan target detection + secondary scan at target locations**					
**AutoScanJ**<br>++ / ++ / ++	µ-Manager<br><br>Leica LAS-AF/X	ImageJ	2D Fixed (proj)<br>2D Fixed (proj)<br>2D Live (proj)<br>2D Live (proj)	3D Fixed<br>3D Fixed<br>3D Live<br>3D Live	1) Fixed tiled map<br>(mitotic cells + spermatozoa phenotypes)<br>2) Fixed Blocks map<br>(Cytoo chip single cell on pattern)<br>3) Live Tiles<br>(mitosis onset of any cells)<br>4) Live Blocks<br>(mitosis onset of user defined cells)
**[Tischer 14]**<br>++ / + / +	Leica LAS-AF/X	CellProfiler<br>ImageJ	2D Fixed<br>2D Fixed	3D Fixed<br>3D Live	1) Automatically identify plasmodium parasites and image them at high resolution<br>2) Automatically identify suitable ERES sites and trigger FRAP experiment at these location
**NanoJ-Fluidics [Almada 19]**<br>++ /+ / -	µ-Manager	ImageJ	2D Live	3D Fixed	Monitor cells from different regions of interest and automatically trigger micro-fluidics when a cell enters mitosis (detected from cell rounding)
**Micro-Manager Intelligent Acquisition**<br>++ / + / -	µ-Manager	ImageJ	2D Fixed	2D Fixed	Fixed tiled map, ImageJ macro to perform targets detection

**Micropilot [Conrad 11]** **- / + / -**	Leica SP5<br>Zeiss LSM 510<br>Olympus ScanR[1]<br>PerkinElmer Ultraview ERS[1]<br>µ-Manager[2]	Labview	2D Live	3D Live	Automatically identify mitotic cells from live microscopy and perform, high resolution time lapses of these cells, including FRAP experiments in these cells
**Region mapping (pre-detection from still images followed by high resolution tiling)**					
**Micro-Magellan [Pinkard 16]** **++ / ++ / NR**	µ-Manager	ImageJ	3D Fixed	3D Live	Automatically identify 3D regions of interest from coarse resolution images to image these regions by high-throughput microscopy (e.g. high resolution time-lapse)
**OpenHiCAMM [Booth 18]** **++ / + / NR**	µ-Manager	ImageJ	2D Fixed	2D Fixed	Automatically detect tissue 2D regions from large slide and image these regions at higher resolution by tiling the detected regions
**iMSRC [Carro 15]** **++ / - / NR**	Leica LAS-AF/X	ImageJ	2D Fixed	2D Fixed	Automatically detect tissue 2D regions from large slide and image these regions at higher resolution by tiling the detected regions
**Object tracking (follow objects during time-lapses, possibly trigger photo-manipulation on the objects)**					
**[Peravali 11]** **+ / - / NR**	Olympus ScanR and XML files	Matlab	3D Live	3D Live	Automatically detect 3D regions (Zebrafish brain) and perform higher resolution microscopy of these regions
**[Rabut 04]** **+ / - / NR**	Visual Basic macro for Zeiss Zen Black	Visual basic macro for Zeiss Zen Black	3D Live	3D Live	Automatically track cells imaged from different regions of interest and adjust the imaging position to follow the cells
**[Leifer 11]** **+ / - / NR**	MindControl (Custom C software)	Matlab	3D Live	3D Live	Automatically follow specific regions from freely moving Caenorhabditis elegans and perform optogenetic manipulation at these locations

Table S1 Features of the main open source Intelligent Microscopy and Online Feedback Microscopy software published as scientific articles

[1] Limited support
[2] Untested

References

[Feiguin 97] Feiguin, F., Llamazares, S., & González, C. 16 Methods in Drosophila Cell Cycle Biology. In *Current Topics in Developmental Biology* (pp. 279–291). Elsevier (1997).

[Rabut 04] Rabut, G., Ellenberg, J. Automatic real-time three-dimensional cell tracking by fluorescence microscopy. Journal of Microscopy, 216, 131–137 (2004).

[Sarrate 09] Sarrate Z, Anton E. Fluorescent in situ hybridization (FISH) protocol in human sperm. *J Vis Exp.* 31 (2009).

[Conrad 11] Conrad, C., A. Wunsche, T.H. Tan, J. Bulke-scher, F. Sieckmann, F. Verissimo, A. Edelstein, T. Walter, et al. Micropilot: automation of fluorescence microscopy-based imaging for systems biology. Nat. Methods 8:246-249 (2011).

[Leifer 11] Leifer, A. M., Fang-Yen, C., Gershow, M., Alkema, M. J., & Samuel, A. D. T. (2011). Opto-genetic manipulation of neural activity in freely moving Caenorhabditis elegans. Nature Methods, 8, 147–152.

[Peravali 11] Peravali, R., Gehrig, J., Giselbrecht, S., Lütjohann, D. S., Hadzhiev, Y., Müller, F., et al. Automated feature detection and imaging for high-resolution screening of zebra-fish embryos. BioTechniques, 50, 319–324 (2011).

[Sdelci 12] Sdelci, Sara, Martin Schütz, Roser Pinyol, M. Teresa Bertran, Laura Regué, Carme Caelles, Isabelle Vernos, and Joan Roig. Nek9 phosphorylation of NEDD1/GCP-WD contributes to Plk1 control of γ-tubulin recruitment to the mitotic centrosome. *Current Biology* 22, no. 16: 1516-1523 (2012).

[Gogo 13] Godo A, Blanco J, Vidal F, Anton E. . Accumulation of numerical and structural chromosome imbalances in spermatozoa from reciprocal translocation carriers. *Hum. Reprod* 28:840–849 (2013).

[Tischer 14] Tischer C, Hilsenstein V, Hanson K, Pepperkok R. Adaptive fluorescence microscopy by online feedback image analysis. Methods in Cell Biology; 123:489-503 (2014).

[Carro 15] Carro, A., Perez-Martinez, M., Soriano, J. et al. iMSRC: converting a standard automated microscope into an intelligent screening platform. Scientific Reports 5, 10502 (2015).

[Pinkard 16] Pinkard H, Stuurman N, Corbin K, Vale R, Krummel MF. Micro-Magellan: open-source, sample-adaptive, acquisition software for optical microscopy. Nature Methods. Sep;13(10):807-809 (2016).

[Booth 18] Booth BW, McParland C, Beattie K, et al. OpenHiCAMM: High-Content Screening Software for Complex Microscope Imaging Workflows. Iscience. Apr;2:136-140 (2018).

[Leica 20] Retrieved October 13, 2020, from

https://www.leica-microsystems.com/products/microscope-accessories/p/leica-hcs-a/
